# Cordycepin stimulates autophagy in macrophages and prevents atherosclerotic plaque formation in ApoE^-/-^ mice

**DOI:** 10.18632/oncotarget.21886

**Published:** 2017-10-16

**Authors:** Xin Li, Yue Zhou, Xue Zhang, Xiaoxue Cao, Chongming Wu, Peng Guo

**Affiliations:** ^1^ Pharmacology and Toxicology Research Center, Institute of Medicinal Plant Development, Chinese Academy of Medical Sciences & Peking Union Medical College, Beijing 100193, P.R. China

**Keywords:** cordycepin, atherosclerosis, foam cell, autophagy, AMPK

## Abstract

Autophagy in macrophages plays a key role in the pathogenesis and progression of atherosclerosis and has become a potential therapeutic target. Here we show that cordycepin (Cpn), a natural derivative of adenosine, markedly reduced atherosclerotic plaque and ameliorated associated symptoms such as dyslipidemia, hyperglycemia and inflammation in ApoE^-/-^ mice. Supplementation of Cpn dose-dependently inhibited oxLDL-elicited foam cell formation and modulated intracellular cholesterol homeostasis by inhibiting cholesterol uptake and promoting cholesterol efflux in RAW264.7 macrophages. Notably, Cpn exhibited significant stimulating effect on macrophage autophagy, as estimated by western blotting, immunofluorescent staining and autophagic vacuoles observation by transmission electron microscopy. The inhibitive effects of Cpn on foam cell formation were dramatically deteriorated in the presence of various autophagy inhibitors, suggesting that autophagy participate, at least in part, in the atheroprotective role of Cpn. Further investigations using different autophagy inhibitors and specific siRNAs for AMP-activated protein kinase (AMPK) gamma1 subunit indicated that Cpn may stimulate macrophage autophagy through AMPK-mTOR pathway. Together, our results demonstrated Cpn as a potential therapeutic agent for the prevention and treatment of atherosclerosis, and the autophagic activity presents a novel mechanism for Cpn-mediated atheroprotection.

## INTRODUCTION

Atherosclerosis (AS), a chronic metabolic and inflammatory disease characterized by atheromatous plaques in the intima of the large arteries, is a major cause of mortality and morbidity worldwide [1]. Growing data have shown that the internalization of modified low-density lipoprotein (LDL), such as oxidized LDL (oxLDL), by macrophages is the crucial process in the development of AS, which triggers foam cell formation and leads to the inflammatory milieu and atherosclerotic plaque growth [2]. The homeostasis of lipids in macrophages is regulated by both the uptake of modified lipoproteins and the efflux of intracellular cholesterol. The scavenger receptors SR-A, SR-B and CD36 bind and internalize ox-LDL while ATP-binding cassette transporters A1/G1 (ABCA1/ABCG1) and their upstream regulators liver X receptor α (LXRα) and peroxisome proliferator-activated receptor γ (PPARγ) promotes the efflux of cholesterol [3, 4]. Nowadays, enhancing cholesterol efflux from macrophages and foam cells via upregulation of PPARγ-LXRα-ABCA1/G1 pathway is considered atheroprotective.

Currently, control of the incidence and progress of AS is still challenging because the involved molecular mechanisms remain incompletely understood. Recently, a growing body of evidence suggests that autophagy, a highly conserved catabolic pathway which transports intracellular components to lysosomes for degradation and recycling, is a critical modulator in the development of AS [5]. Many studies have shown that basal autophagy in macrophages plays a key role in preventing foam cell formation and atherosclerosis incidence [6]. Disruption of macrophage autophagy leads to marked development of atherosclerotic plaques in mice [7, 8]. The proposed mechanisms include decreases in lipophagy, increased inflammation, and enhanced cell death [5]. Under atherogenic conditions, the accumulated oxLDL and reactive oxygen species may result in disrupted autophagy, which exacerbates atherosclerosis. Lipophagy is the autophagic removal of lipids, which contributes to macrophage cholesterol efflux [8]. Induction of autophagy in macrophages by berberine and 3-methyladenine promotes the breakdown of the intracellular lipids and the delivery of cholesterol esters to lysosomes for cholesterol efflux, thereby preventing foam cell formation and atherosclerotic plaque development [9, 10]. Therefore, enhancing macrophage autophagy represents a promising approach to deal with atherogenesis.

Cordycepin (3’-deoxyadenosine, hereafter named Cpn) is a natural derivative of adenosine, possessing multiple pharmacological activities including anti-oxidation, anti-tumor, anti-inflammation [11], neuroprotection [12] and preventing bone loss [13]. We and other groups have shown that Cpn is also effective to ameliorate metabolic disorders, such as dyslipidemia, insulin resistance and type 2 diabetes [14, 15]. Recent studies demonstrated that the anti-hyperlipidemic effect of Cpn may attribute to the activation of AMPK [16]. As dyslipidemia and inflammation are both key factors for the development of atherosclerosis, the anti-inflammatory and dyslipidemia-improving effects of Cpn mark it a promising agent for the prevention and treatment of atherosclerosis. However, the atherosclerosis-attenuating effect of Cpn is still lack of proof and the potential mechanism remains largely unknown.

In the present study, we ascertained the anti-atherosclerotic effects of Cpn in high-fat diet (HFD)-fed ApoE-/- mice and proved that Cpn is efficacious to prevent foam cell formation in macrophages by modulating intracellular cholesterol homeostasis. Notably, we found Cpn is a potent agonist of macrophage autophagy and the autophagic activity plays a key role in Cpn-mediated atheroprotection. Our results provide novel insights for the mechanisms of Cpn in the prevention and treatment of atherosclerosis.

## RESULTS

### Cordycepin prevents the development of atherosclerosis in apoE^-/-^ mice

**Figure 1:**
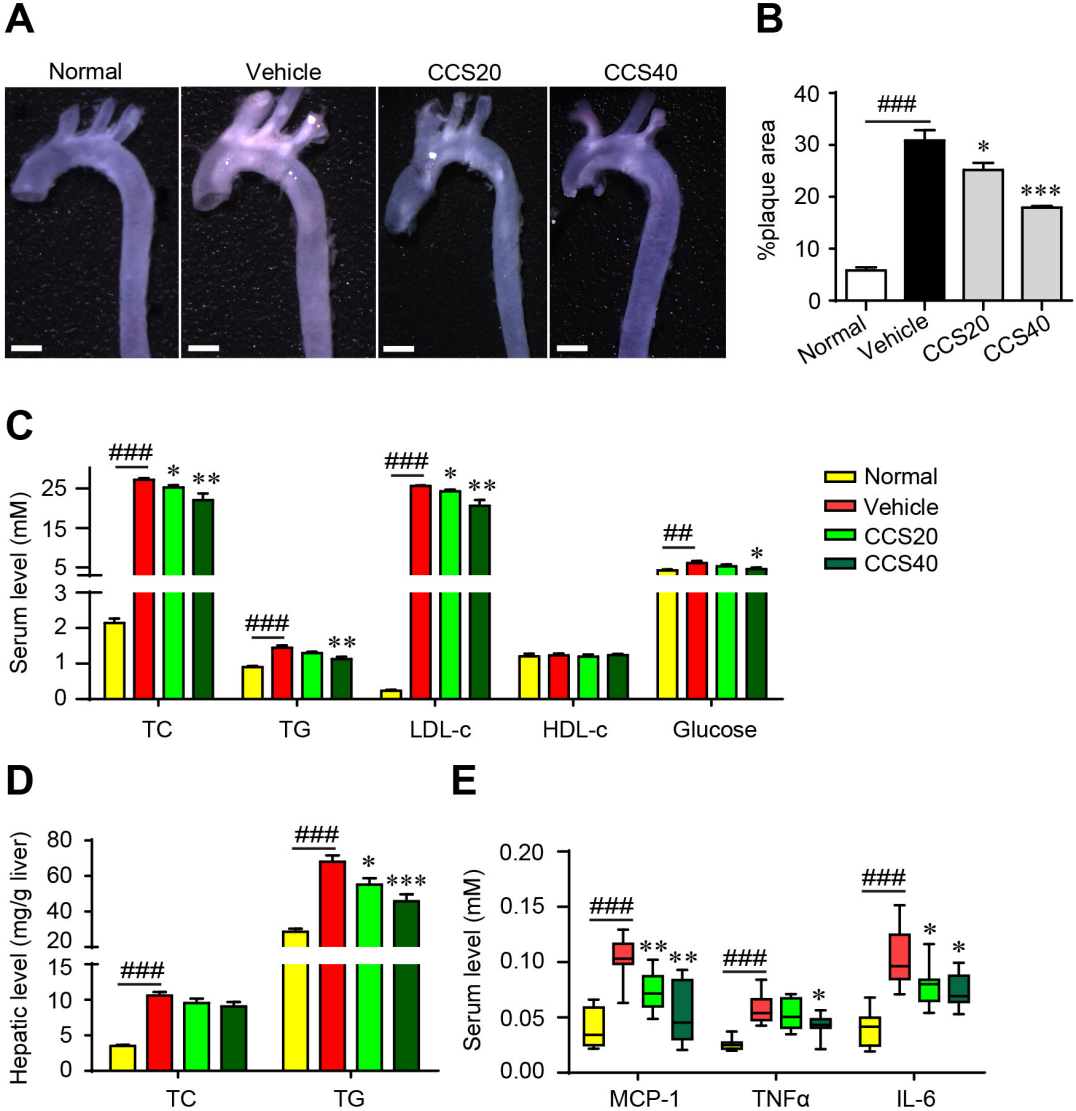
Cordycepin (Cpn) inhibits atherosclerotic lesion formation **(A)** Representative image of the whole aorta. Bar = 1 mm. **(B)** Quantitation of atherosclerotic lesions in the whole aorta. **(C)** Serum lipids and glucose. **(D)** Hepatic levels of TC and TG. **(E)** Serum levels of pro-inflammatory factors. Values are means ± SEM (n = 8). ^##^*p* < 0.01, ^###^*p* < 0.001, vehicle vs. normal, ^*^p < 0.05, ^**^p < 0.01, ^***^p < 0.001, test group vs. vehicle group. TC, total cholesterol; TG, triglycerides; LDL-c, low-density lipoprotein cholesterol; HDL-c, high-density lipoprotein cholesterol; MCP-1, monocyte chemotactic protein-1; TNFα, tumor necrosis factor α; IL-6, interleukin-6.

To evaluate the potential inhibitive effect of Cpn on the development of atherosclerotic plaques, apoE^-/-^ mice were fed with high fat-diet (HFD) and treated with or without Cpn for 16 weeks. As compared with the C57BL/6 normal control, HFD-fed apoE^-/-^ mice displayed multiple atherosclerotic lesions in the aorta arch (Figure 1A), revealing remarkable atherosclerosis. In contrast, treatment with Cpn significantly and dose-dependently reduced atherosclerotic lesions (Figure 1A), indicating a potent anti-atherosclerotic effect.

The atherosclerotic mice were accompanied with marked dyslipidemia and hyperlipidemia, as revealed by significant increase in serum levels of total cholesterol (TC), triglycerides (TG), low-density lipoprotein cholesterol (LDL-c) and glucose as well as increased levels of liver TC and TG (Figure 1C and 1D). Administration of Cpn dose-dependently ameliorated all the parameters for dyslipidemia and hyperlipidemia (Figure 1C and 1D), which was in accordance with previous reports [14–16]. Simultaneously, the increased levels of serum monocyte chemotactic protein-1 (MCP-1), tumor necrosis factor α (TNFα) and interleukin-6 (IL-6) in HFD-fed apoE^-/-^ mice were also largely suppressed (Figure 1E). These data suggest that Cpn is effective to eliminate multiple risk factors and thus prevent the incidence and progress of atherosclerosis

### Cordycepin inhibits foam cell formation by regulating intracellular cholesterol homeostasis

**Figure 2:**
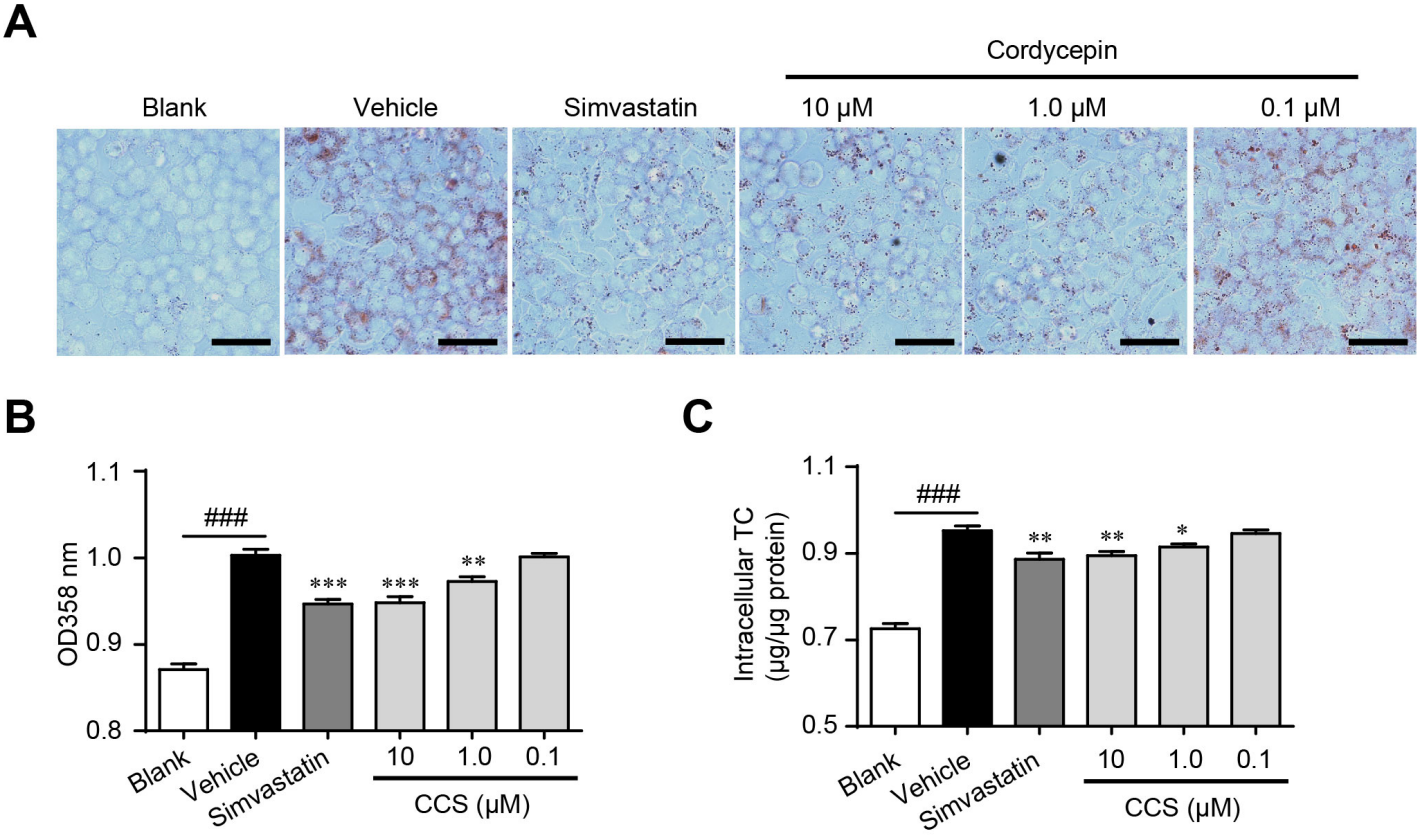
Cordycepin (Cpn) inhibited oxidized low-density lipoprotein (oxLDL)-elicited foam cell formation in RAW264 7 cells. RAW264.7 cells were elicited by oxLDL for 24 h with or without supplementation of Cpn or simvastatin. Cells were then stained with oil-red O, and the representative staining pictures **(A)**, the absorptance at 358 nm **(B)**, and intracellular total cholesterol content **(C)** were acquired. Bar = 50 μm. Values are means ± SEM of at least three experiments. ^###^*p* < 0.001, vehicle vs. blank, ^*^p < 0.05, ^**^p < 0.01, ^***^p < 0.001, test group vs. vehicle group.

Lipid deposition in macrophage induces foam cell formation and ultimately accelerates the development of atherosclerosis [6]. Although the inhibitive actions of Cpn on dyslipidemia and inflammation had been demonstrated [15–17], its effects on foam cell formation have not been reported. We determined oxLDL-induced foam cell formation by using oil-red O staining and intracellular TC quantification. Supplemented with Cpn (0.1-10 μM) significantly decreased oxLDL-elicited lipids deposit and TC accumulation in RAW264.7 macrophages (Figure 2), showing clear inhibiting effects on foam cell formation. The inhibiting efficacy of Cpn (10 μM) was comparable to that of simvastatin, a popular drug for the prevention and treatment of atherosclerosis. These results suggested that inhibition of macrophage-derived foam cell formation may participate in the preventing effect of Cpn on atherosclerosis development.

**Figure 3:**
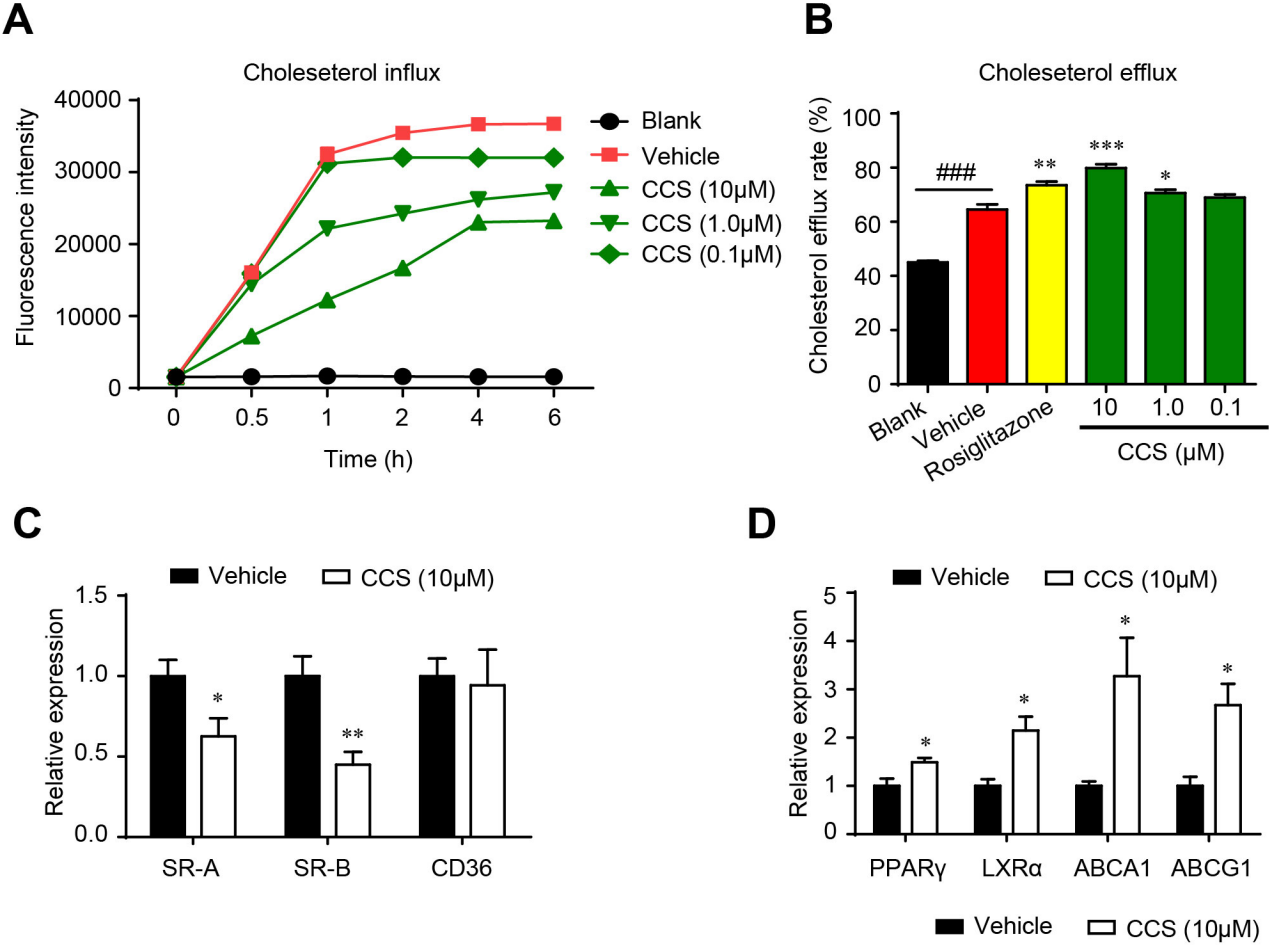
Cordycepin (Cpn) regulated intracellular cholesterol homeostasis by inhibiting cholesterol uptake **(A)**, promoting cholesterol efflux **(B)** and modulating mRNA levels of genes that involved in cholesterol uptake **(C)** and efflux **(D)** in RAW264. 7 macrophages. Values are means ± SEM of at least three experiments. ^###^*p* < 0.001, vehicle vs. blank, *p < 0.05, **p < 0.01, ***p < 0.001, test group vs. vehicle group. SR-A/B, scavenger receptors A/B; CD36, cluster of differentiation 36; PPARγ, peroxisome proliferator-activated receptor γ; LXRα, liver X receptor α; ABCA1/G1, ATP-binding cassette transporters A1/G1.

The cholesterol homeostasis is a key regulator in the development of foam cells. Inhibiting cholesterol uptake and stimulating cholesterol efflux are two effective approaches to suppress foam cell formation. We assessed the effect of Cpn on the cholesterol homeostasis in macrophages using NBD-cholesterol-based fluorescence assays. As shown in Figure 3A, Cpn remarkably inhibited cholesterol uptake by RAW264.7 macrophages in a dose- and time-dependent manner. Simultaneously, Cpn significantly promoted cholesterol efflux from macrophages with an efficiency higher than that of rosiglitazone, a PPARγ agonist that is known to promote cholesterol efflux (Figure 3B). Realtime quantitative PCR also shown that Cpn switched the mRNA levels of cholesterol-uptaking genes (SR-A and SR-B) and cholesterol-effluxing genes (PPARγ, LXRα, ABCA1 and ABCG1) to the intracellular cholesterol-reducing side (Figure 3C and 3D), indicating that Cpn might prevent foam cell formation through regulating cholesterol uptake and efflux.

### Cordycepin promotes autophagy in macrophages

**Figure 4:**
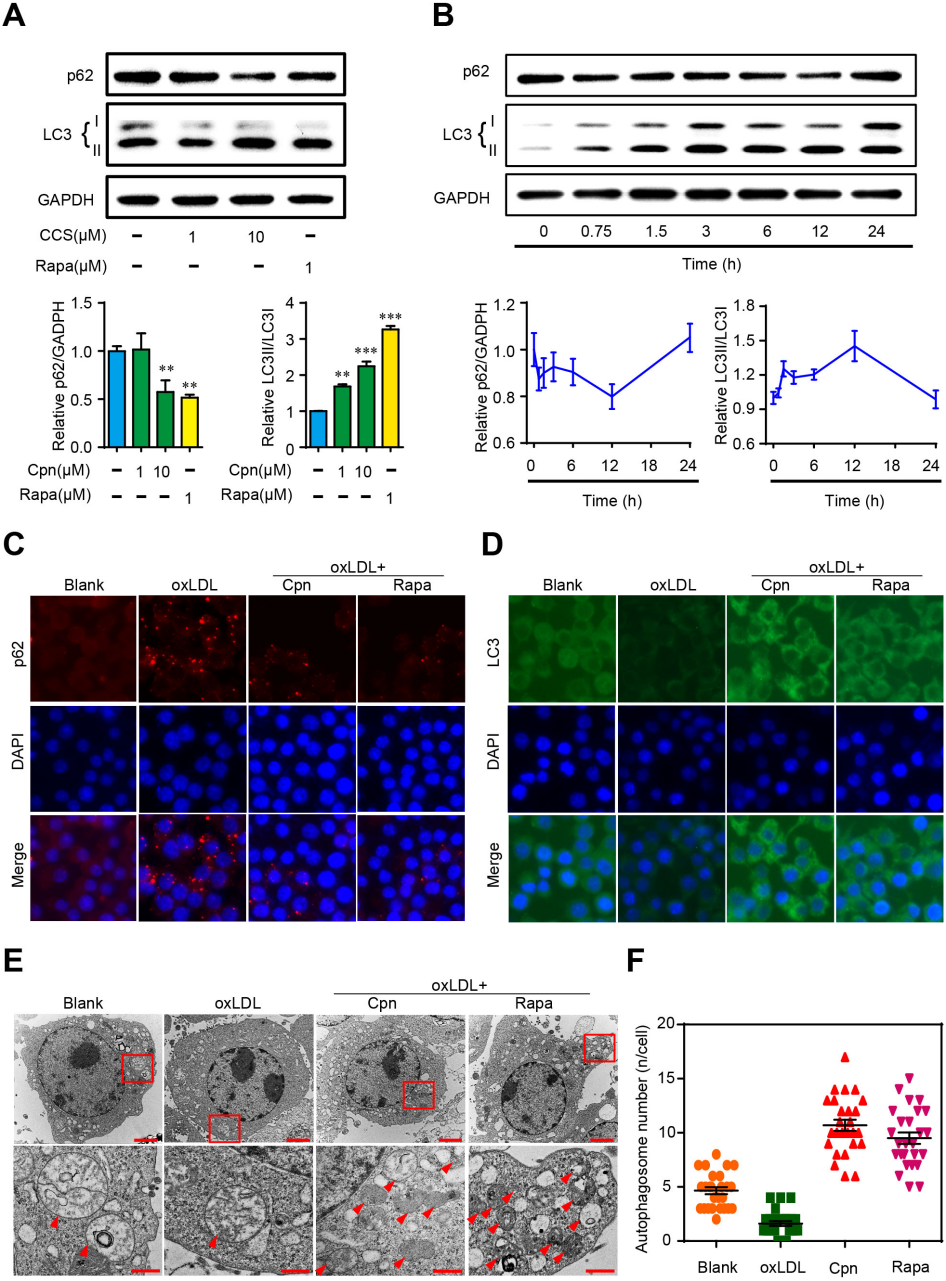
Cordycepin (Cpn) promoted macrophage autophagy **(A)** Western blot analysis of LC3 and p62 levels. **(B)** Western blot analysis of autophagy activity at different times after Cpn stimulation. **(C** and **D)** Immunofluorescence staining of LC3 (D) and p62 (D). **(E)** Ultrastructural changes in RAW264.7 macrophages were observed by TEM. Red arrows indicate autophagosomes. Bar = 2 μm in upper icons and Bar = 500 nm in lower icons. **(F)** Quantitative analysis of autophagosome number in each cell. Values are means ± SEM of at least three experiments. ^**^p < 0.01, ^***^p < 0.001.

Recent studies have shown that autophagy is beneficial to promoting cholesterol efflux and inhibiting foam cell formation in macrophages [8]. To evaluate the effect of Cpn on macrophage autophagy, we first investigated the expression of two commonly used autophagy hallmarks, LC3 (an autophagosome coat protein and a direct marker of autophagy progression) and p62 (a selective autophagy chaperone that detects and delivers large biomolecules such as protein aggregates or organelles to autophagosomes) [5]. Western blotting showed that Cpn decreased the ratio of p62/GADPH ratio and increased the ratio of LC3-II/LC3-I in a dose- and time-dependent manner in RAW264.7 macrophages. The efficacy of 10 μM Cpn was comparable to that of 1 μM rapamycin, and the highest autophagy-promoting effect occurred at 12 h (Figure 4A and 4B). In light of this, we used 10 μM Cpn and 12 h treatment period in the following experiments.

We further assessed the expression of LC3 and p62 by immunofluorescence assay. As shown in Figure 4C and 4D, oxLDL remarkably increased the immunofluorescent signals of p62 and decreased LC3 as compared to the blank control, indicating decreased autophagy in foam cells. Supplementation with Cpn or rapamycin essentially reversed the expression levels of p62 and LC3, which was in accordance with the western blotting results. These results are indicative of autophagy-stimulating activity of Cpn in RAW264.7 cells.

Additionally, we also visualized the autophagic vacuoles by transmission electron microscopy (TEM) after Cpn treatment. TEM results showed that Cpn increased the formation of cytoplasmic vacuoles, often engulfing cellular components or organelles (Figure 4E). We quantified the autophagosome number in individual cell and found that oxLDL decreased the autophagosome number while Cpn significantly increased autophagosome in macrophages (Figure 4F). Together, our data clearly indicate that Cpn stimulates autophagy in RAW264.7 macrophages.

### Autophagy plays a key role in the anti-foam cell formation effect of cordycepin

**Figure 5:**
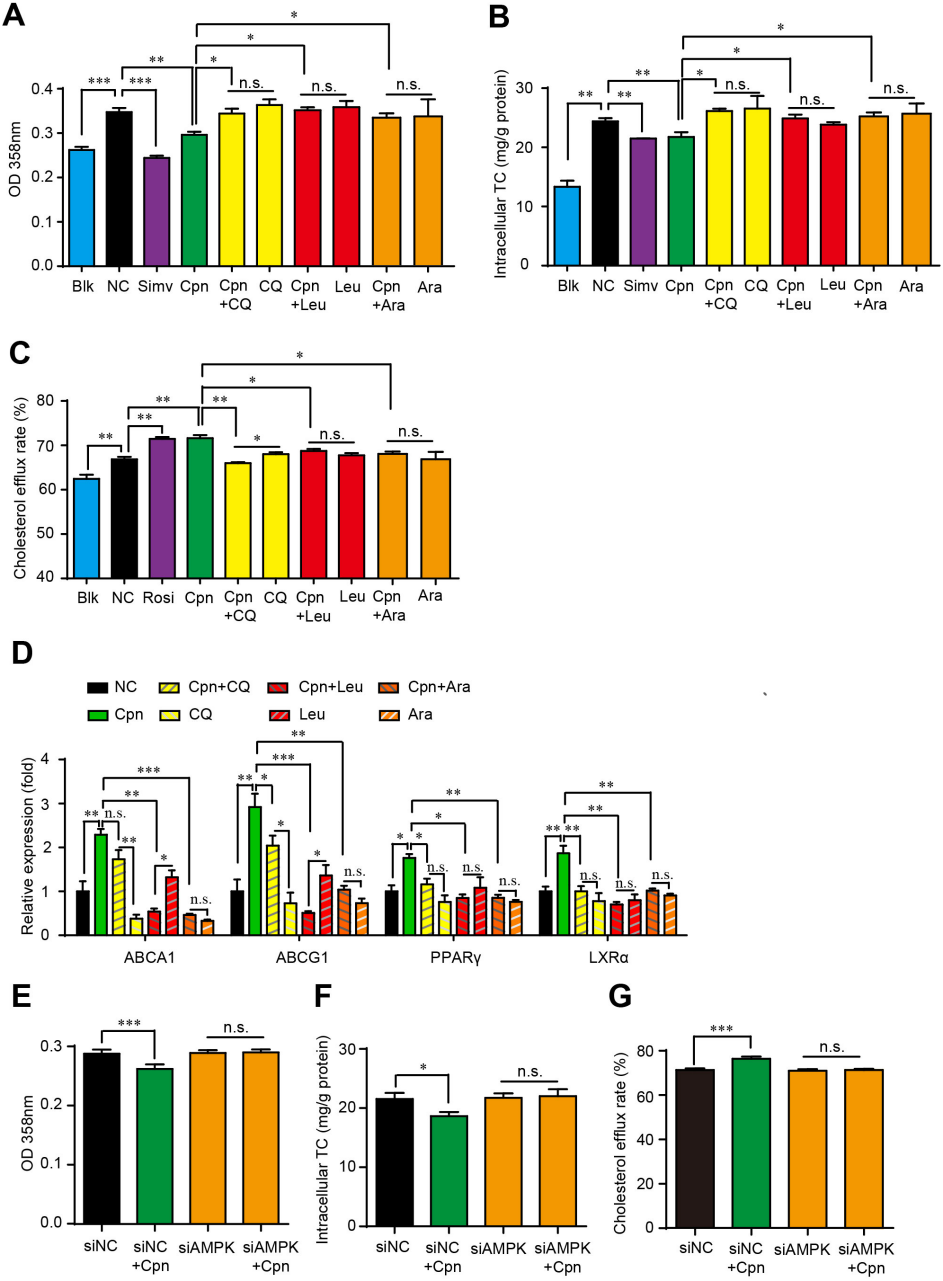
Autophagic activity played a key role in Cpn-elicited anti-foam cell formation effect **(A)** and **(B)** Absorptance at 358 nm after oil-red O staining. **(C)** and **(D)** Intracellular TC levels. **(E)** and **(F)** Cholesterol efflux rate. **(G)** mRNA levels of genes that promote cholesterol efflux. Values are means±SEM of at least three experiments. *p < 0.05, **p < 0.01, ***p < 0.001.

To verify whether the autophagic activity of Cpn is essential for its anti-atherosclerotic effect, we interfered macrophage autophagy with various inhibitors and assessed their influence on the regulating effects of Cpn on foam cell formation. As estimated by oli-red O staining and intracellular TC quantification, autophagy inhibitors, chloroquine (CQ), leucine (Leu) and 9-β-d-arabinofuranosyl adenine (Ara-A) were all able to essentially abolish the inhibitive effects of Cpn on intracellular lipid accumulation (Figure 5A) and TC content (Figure 5B). The simulating effects of Cpn on cholesterol efflux rate (Figure 5C) and expression levels of cholesterol effluxing genes such as PPARγ, LXRα, and ABCA1/G1 (Figure 5D), were also largely deteriorated by all the three autophagy inhibitors. At the same time, the modulating effects of Cpn on intracellular lipid accumulation and cholesterol efflux rate were substantially abolished when AMPKγ1 subunit was knocked-down by specific siRNAs (Figure 5E-5G). These results indicated that autophagy, especially the autophagic regulator AMPK, plays a key role in the anti-atherosclerotic effect of Cpn.

### Cordycepin stimulates autophagy via AMPK-mTOR pathway in macrophages

**Figure 6:**
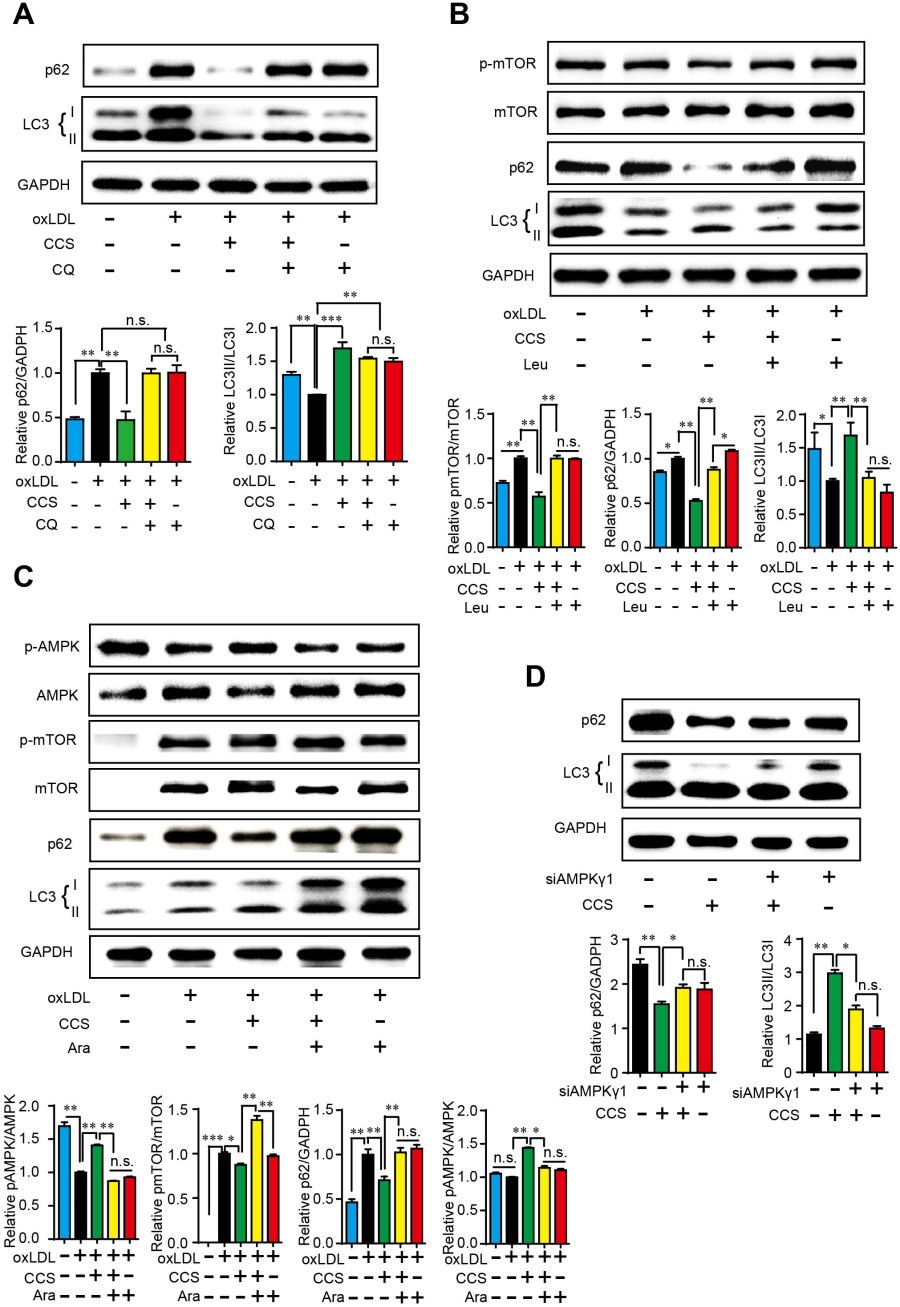
Cpn stimulated macrophage autophagy through AMPK-mTOR pathway Western blot analysis showed that late phase autophagy inhibitor chloroquine (CQ) **(A)**, mTOR activator leucine **(B)** and AMPK inhibitor (Ara-A) **(C)** largely abolished the regulating effect of Cpn on macrophage autophagy. **(D)** Specific siRNAs for AMPKγ1 subunit significantly deteriorated the regulating effect of Cpn on macrophage autophagy. Values are means±SEM of at least three experiments. ^*^p < 0.05, ^**^p < 0.01, ^***^p < 0.001. n.s. = nonsignificant.

To explore the potential mechanism though which Cpn stimulates autophagy, we tested the influence of several known autophagy regulators on Cpn-elicited autophagy in macrophages. Chloroquine (CQ) is a lysosome inhibitor which can up-regulate LC3-II by inhibiting the fusion of autophagosomes and lysosomes [18]. As shown in Figure 6A, LC3-II was remarkably aggregated by CQ, which was in accordance with previous report. Cpn dramatically decreased p62 and increased LC3-II/LC3-I ratio but the autophagic effect of Cpn turned insignificant when co-treated with CQ (oxLDL+Cpn+CQ vs oxLDL+ CQ). Similarly, addition of leucine resulted in increased mTOR phosphorylation and decreased autophagy as reported previously [19]. The regulating effects of Cpn on p62 and LC3-II/LC3-I ratio were substantially abolished in presence of leucine (Figure 6B), suggesting that mTOR may participate in the autophagic activity of Cpn.

AMPK-mTOR is a key regulating pathway of autophagy [20] and we have demonstrated that Cpn is a potent AMPK activator [16]. In this study, we tested the involvement of AMPK in Cpn-mediated autophagy by two approaches. First, we used AMPK inhibitors to interfere the autophagic role of Cpn. Since the most commonly used AMPK inhibitor, compound C, can stimulate autophagy through AMPK-independent pathway [21], we took an alternative AMPK inhibitor, Ara-A, in our study. As shown in Figure 6C, Ara-A decreased the phosphorylation of AMPK and increased mTOR phosphorylation, which resulted in reduced autophagy in macrophages as revealed by increased p62 and decreased LC3-II/LC3-I ratio. Interestingly, the modulating effects of Cpn on AMPK, mTOR, p62 and LC3-II/LC3-I ratio were all largely reversed in the presence of Ara-A, indicating that Cpn might promote autophagy through AMPK-mTOR pathway. To further confirm the essential role of AMPK in the autophagic role of Cpn, we down-regulated the expression of AMPK gamma1 subunit by specific siRNAs. The autophagic effect of Cpn was largely reduced when AMPK gamma1 subunit was knocked-down (Figure 6D). These results indicated that Cpn might stimulate autophagy via AMPK-mTOR pathway in macrophages.

## DISCUSSION

Finding natural products with atheroprotective activities is essential for the development of new drugs to prevent and cure atherosclerosis. Recent studies have demonstrated that macrophage autophagy plays a key role in preventing atherosclerosis [5, 8]. In this work, we identified cordycepin (Cpn) as an effective compound to treat atherosclerosis. Importantly, we demonstrate that promoting macrophage autophagy represents a novel mechanism of Cpn in inhibiting foam cell formation and preventing atherosclerosis development.

Cordycepin (Cpn) is a natural derivative of adenosine possessing multiple activities. Previously, we and other groups have demonstrated that Cpn is effective to ameliorate metabolic disorders, such as decreasing blood lipids, improving insulin resistance and alleviating chronic inflammation [15, 16, 22], which marks Cpn as a potential therapeutic agent for atherosclerosis. Recently, Won et al reported that Cpn could attenuate neointimal formation induced by balloon-injury in rats [22]. However, the rat carotid artery balloon injury model is established by mechanical damage, which is different from the actual development of atherosclerosis. In this work, we confirmed the atheroprotective effects of Cpn in classical HFD-fed apoE^-/-^ atherosclerotic mice. Our results showed that oral administration of Cpn for 16 weeks significantly and dose-dependently reduced atherosclerotic lesions in apoE^-/-^ mice, revealing a potent atheroprotective activity. The key risk factors for the development of atherosclerosis such as dyslipidemia and inflammation were all significantly reduced after treatment with Cpn. Besides, Cpn markedly decreased oxLDL-elicited foam cell formation in macrophages as determined by oil-red O staining and intracellular TC quantification. Fluorescence-based cholesterol uptake and efflux assays and realtime quantitative PCR revealed that Cpn might prevent foam cell formation through inhibiting cholesterol uptake and stimulating cholesterol efflux. Together, these data proved that Cpn is a promising candidate for the treatment of atherosclerosis and it can prevent and cure atherosclerosis through multiple ways.

An important finding in this work is that Cpn can markedly stimulate macrophage autophagy. Three sets of experiments were performed to assess the promoting effects of Cpn on macrophage autophagy. First, we checked the expression levels of two autophagy hallmarks, LC3 and p62, by western blotting. Treatment with Cpn dose- and time-dependently decreased the ratio of p62/GADPH and increased the ratio of LC3-II/LC3-I. The efficacy of 10 μM Cpn was comparable to that of 1 μM rapamycin, indicating potent autophagic activity. We further assessed the effect of Cpn on LC3 and p62 by immunofluorescence approach. The immunofluorescent signals showed that oxLDL remarkably increased the staining of p62 and decreased LC3 while Cpn substantially reversed the expression of p62 and LC3, indicating remarkable autophagy-stimulating activity. Lastly, we observed the autophagic vacuoles by transmission electron microscopy. The TEM photographs revealed that Cpn significantly increased the autophagosome number in macrophages. These results clearly proved that Cpn can potently promote autophagy in RAW264.7 macrophages.

We further assessed whether macrophage autophagy was essential for the anti-atherosclerotic activity of Cpn. To make a thorough evaluation about the influence of autophagy on Cpn-mediated anti-atherosclerotic activity in macrophages, three common inhibitors that act on different stage of autophagy were used and the cells were evaluated from four aspects, that were, oil-red O staining, intracellular TC quantification, cholesterol efflux and mRNA levels of atheroprotective genes. Our results displayed that all three autophagy inhibitors can substantially deteriorate the inhibitive effects of Cpn on foam cell formation as estimated by oil-red O staining and intracellular TC quantification. The regulating roles of Cpn on cholesterol efflux and mRNA levels of genes that involve in cholesterol efflux were also degraded in the presence of the three autophagy inhibitors. These data provided obvious evidence that the autophagic activity played a key role in the anti-atherosclerotic activity of Cpn.

To explore the potential mechanism of Cpn-mediated autophagic effect, we used three inhibitors that act on different stage of autophagy. Chloroquine (CQ) interferes the fusion of autophagosomes and lysosomes, thus inhibiting the final stage of autophagy [18]. Leucine (Leu) is an activator of mTOR and inhibits the core procedure of autophagy [19]. And Ara-A is an antagonist of AMPK which functions at the upstream of autophagy [23]. Western blotting showed that both CQ and Leu were able to inhibit the modulatory effect of Cpn on p62/GAPDH and LC3-II/LC3-I ratios, suggesting that Cpn might activate macrophage autophagy at the upper stream. As AMPK-mTOR is a key regulating pathway of autophagy [20] and we have demonstrated that Cpn is a potent AMPK activator [16]. We further investigated the involvement of AMPK in Cpn-mediated autophagy. First, we took used of AMPK inhibitor, Ara-A, to interfere macrophage autophagy and observed its impact on the autophagic effect of Cpn. Our results showed that supplementation of Ara-A largely reversed the modulating effects of Cpn on AMPK, mTOR, p62 and LC3-II/LC3-I ratio, suggesting that AMPK plays a key role in Cpn-mediated autophagic effect. To further confirm this hypothesis, AMPK gamma1 subunit was knocked-down by specific siRNAs and the autophagic effect of Cpn was largely reduced. These results indicated that Cpn might stimulate autophagy via AMPK-mTOR pathway.

## MATERIALS AND METHODS

### Ethics statement

All the animal experiments were performed in accordance with the National Institutes of Health regulations for the care and use of animals in research and were approved by the Medical Ethics Committee of Peking Union Medical College (No. YZS201604014).

### Animal experiment

Apolipoprotein E (ApoE) is a ligand for lipoprotein recognition and clearance by lipoprotein receptors. ApoE-deficient mice (ApoE-/- mice) have delayed clearance of lipoproteins and develop widespread fibrous plaque lesions at vascular sites typically affected in human atherosclerosis [24]. In our study, eighteen male ApoE-/- mice (8 weeks old, 18 - 23 g) and six male C57BL/6 mice (8 weeks old, 18 - 21 g) were purchased from Vital River Laboratory Animal Technology Co., Ltd. (Beijing, China). Animals were kept in a temperature-controlled (24°C) room under a 12 h/12 h day/night cycle with food and water available *ad libitum*. After acclimation for 1 week, mice were randomly divided into four groups with six animals in each group. The normal group (C57BL/6 mice) was fed with D12450B normal diet chow and the other groups (ApoE^-/-^ mice) were fed with D12492 HFD. The negative control group was given equal volumes of vehicle (distilled water) while Cpn20 and Cpn40 groups were treated with 20 mg/kg/day and 40 mg/kg/day of Cpn (*p.o.*), respectively. At the end of the 16-week period, the animals were anaesthetised, and blood samples were collected from the abdominal aorta. The periaortic tissue was trimmed around the aorta and the whole aorta was taken. Images were obtained with a Zeiss Axio Camera (CarlZeiss, Jena, Germany). The plaque area of the total vessel surface area was measured using ImageJ software. A piece of liver tissue was excised and homogenised in saline for the determination of hepatic TC and TG. Biochemical parameters were measured with respective kits according to the manufacturer’s instruction. The kits for TC (Lot. F002-2), TG (Lot. F001-2), LDL-c (Lot. F004-2), HDL-c (Lot. F003-2), glucose (Lot. F006), TNFα (Lot. H052), IL-6 (Lot. H007) and MCP-1(Lot. H115) were purchased from Jian Cheng Biotechnology Company, Nanjing, China.

### Cell culture and foam cell formation

Murine RAW264.7 cells (Peking Union Medical College, China) were cultured in Dulbecco’s modified Eagle medium (DMEM) supplemented with 10% fetal bovine serum, penicillin and streptomycin at 37 °C in 5% CO_2_ atmosphere. When grown to 70% - 80% confluence, cells were incubated in DMEM supplemented with 50 μg/ml oxLDL (Xiesheng Biotechnologies, Beijing, China) and respective agents (distilled water, or simvastatin, 10 μM, or Cpn, 10 - 0.1 μM as indicated) for 24 hours. Cells without oxLDL treatment were used as a blank control. Subsequently, the cells were subjected to oil-red O staining or total cholesterol determination as described previously [25].

### 25-NBD cholesterol uptake assay

25-NBD cholesterol was used as indicator to monitor cholesterol uptake by RAW264.7 macrophages. The cells were cultured in 96-well black plates (Costar, Corning Inc., Corning, NY, USA) at 4 x 10^4^ cells per well for 6 h. Then cells were transferred into serum-free DMEM containing 5 μg/mL 25-NBD cholesterol and different concentration of Cpn for indicated time. Afterwards, cells were washed twice with phosphate buffered saline (PBS), and the fluorescence intensity of intracellular 25-NBD cholesterol was measured using a Tecan Infinite M1000Pro Microplate Reader (TECAN Group Ltd., Shanghai, China; excitation 485 nm, emission 535 nm). Each uptake assay was performed in triplicate with 3 replicates each.

### Cholesterol efflux assay

RAW264.7 cells were equilibrated with 25-NBD cholesterol (1 mg/mL) for 12 h. NBD-cholesterol-labeled macrophages were washed twice with PBS and incubated in serum-free DMEM medium containing 50 mg/mL HDL and respective agents (distilled water, or rosiglitazone, 10 μM, or Cpn, 10 - 0.1 μM as indicated) for 6 h. The fluorescence-labeled cholesterol released from cells into the medium was measured with a Tecan Infinite M1000Pro Microplate Reader. Cholesterol efflux was expressed as a percentage of fluorescence in the medium relative to the total amounts of fluorescence detected in cells and the medium. Each experiment was performed in triplicate with 3 replicates each.

### Real-time PCR

Total RNA extraction, cDNA synthesis and quantitative PCR assays were performed as previously reported [25]. At least three independent biological replicates were performed to verify the reproducibility of the data. The gene-specific primers used for quantitative PCR are listed in Supplementary Table 1. Data are presented as fold change in the experimental group normalized to actin and relative to the control group.

### Western blot

Cultured cells were extracted by lysis buffer containing 10% glycerol, 1% Triton X-100, 135 mM NaCl, 20 mM Tris (pH 8.0), 2.7 mM KCl, 1 mM MgCl_2_, and protease and phosphatase inhibitors (0.5 mM PMSF, 2 μM pepstatin and 2 μM okadaic acid). Aliquots of samples were separated on 12% SDS-ployacrylamide gel electrophoresis and transferred onto polyvinylidene difluoride (PVDF) membranes (Amersham Pharmacia, Uppsala, Sweden). The membranes were blocked with 10% (wt/vol) skimmed milk in Tris-buffered saline solution containing 0.1% Tween-20 for 1 h. Immunoblotting was performed overnight at 4°C with respective primary antibodies (1:1000 dilution; all from Cell Signaling Technology), followed by peroxidase-conjugated secondary antibody (Sigma-Aldrich, Shanghai, China) for 1 h at room temperature. Proteins were detected with ECL plus kits (Amersham, Piscataway, NJ, USA). Western blots were prepared at least three times for each sample.

### Autophagosome immunofluorescence

LC3 and P62 proteins are specific markers for autophagosome formation and degradation, respectively. To assess LC3 and P62, cells were cultured on glass coverslips and treated as indicated for 12 h. After fixed in 4% paraformaldehyde for 30 min, the slips were blocked with 10% normal goat serum for 1 h at room temperature. Cells were then incubated with anti-LC3 antibody (1:200, Cell Signaling Technology) or anti-P62 antibody (1:200, Cell Signaling Technology) overnight at 4°C. After incubated with Alexa Fluor 488 goat anti-rabbit IgG (1:200, Invitrogen), the cells were counterstained with 4',6-diamidino-2-phenylindole (DAPI, Sigma) to mark the nuclei. Images were captured with EVOS Cell Imaging Systems (Thermo Fisher Scientific).

### Transmission electron microscopy (TEM)

Cells were centrifuged and fixed with 2.5% glutaraldehyde. After washed with cold PBS, the cells were post-fixed in 1% osmium tetroxide for 1 h at 4°C. Then samples were dehydrated with increasing concentrations of ethanol and embedded in poly/Bed-812 resin. The ultrathin sections were stained with uranyl acetate and photographed with a transmission electron microscope (HITACHI H-7650).

### Transfection of a siRNA targeting AMPKγ1 subunit

Raw264.7 macrophages were transiently transfected with AMPKγ1 siRNAs for 6 h using LipofectAMINE 2000™ (Invitrogen, Carlsbad, CA) according to the manufacturer’s protocol. The medium was then replaced with fresh medium containing 10% FBS and respective agent. After 24 h recovery, cells harvested for mRNA and protein extraction.

### Statistical analysis

Data are expressed as mean ± S.E.M. One-way ANOVA was used to determine significant differences among groups, after which the modified Student’s *t*-test with the Bonferroni correction was used for comparison between individual groups. All statistical analyses were performed with SPSS 17.0 software (SPSS Inc., Chicago, IL, USA). *P* < 0.05 was considered statistically significant.

## CONCLUSION

Our results demonstrated that cordycepin (Cpn) is an effective compound to prevent atherosclerosis plaque formation *in vivo*. Cpn can stimulate macrophage autophagy through AMPK-mTOR pathway, which plays a key role in the anti-atherosclerotic activity of Cpn.

## SUPPLEMENTARY MATERIALS TABLE



## Abbreviations

ABCA1/G1: ATP-binding cassette transporters A1/G1
Ara-A: 9-β-d-Arabinofuranosyl Adenine
AS: Atherosclerosis
Cpn: cordycepin
CQ: chloroquine
DAPI: 4',6-diamidino-2-phenylindole
HFD: high fat-diet
IL-6: interleukin-6
LDL: low-density lipoprotein
LXRα: liver X receptor α
MCP-1: monocyte chemotactic protein-1
oxLDL: oxidized LDL
PPARγ: peroxisome proliferator-activated receptor γ
PVDF: polyvinylidene difluoride
SR: scavenger receptors
TNFα: tumor necrosis factor α
