# Supplementary material for: Cordycepin stimulates autophagy in macrophages and prevents atherosclerotic plaque formation in ApoE-/- mice
Source: Oncotarget. 2017 Oct 16;8(55):94726–37. doi: 10.18632/oncotarget.21886 (PMC5706907; doi:10.18632/oncotarget.21886)
Supplement: Supplementary file 1 [file oncotarget-08-94726-s001.pdf]

# Cordycepin stimulates autophagy in macrophages and prevents atherosclerotic plaque formation in ApoE$^{-/-}$ mice

## SUPPLEMENTARY MATERIALS

**Supplementary Table 1: Oligonucleotide primers used in this work**

| Primers | Sequence (5'-3') |
|---|---|
| PPARγ-F | CATTCTGGCCCACCAACTTC |
| PPARγ-R | TCAAAGGAATGCGAGTGGTCTT |
| LXRα-F | CCTTCCTCAAGGACTTCAGTTACAA |
| LXRα-R | CATGGCTCTGGAGAACTCAAAGAT |
| ABCA1-F | GCGGACCTCCTGGGTGTT |
| ABCA1-R | CAAGAATCTCCGGGCTTTAGG |
| ABCG1-F | AAGGCCTACTACCTGGCAAAGA |
| ABCG1-R | GCAGTAGGCCACAGGGAACA |
| β-actin-F | ACACTGTGCCCATCTACGAG |
| β-actin-R | CAGCACTGTGTTGGCATAGAG |
